# “I pity the TB patient”: a mixed methods study assessing the impact of the COVID-19 pandemic on TB services in two major Indonesian cities and distilling lessons for the future

**DOI:** 10.1136/bmjgh-2023-014943

**Published:** 2024-05-16

**Authors:** Yusuf Ari Mashuri, David Boettiger, Siska Dian Wahyuningtias, Srila Nirmithya Salita Negara, Yanri Wijayanti Subronto, Marco Liverani, Luh Putu Lila Wulandari, Riris Andono Ahmad, Hasbullah Thabrany, Nasser Fardousi, John Kaldor, Ari Probandari, Virginia Wiseman

**Affiliations:** 1 Center for Tropical Medicine, Faculty of Medicine, Public Health and Nursing, Universitas Gadjah Mada, Yogyakarta, Indonesia; 2 Faculty of Medicine, Universitas Sebelas Maret, Surakarta, Indonesia; 3 The Kirby Institute, University of New South Wales, Sydney, New South Wales, Australia; 4 Division of Tropical Medicine and Infectious Diseases, Department of Internal Medicine, Faculty of Medicine, Public Health, and Nursing, Universitas Gadjah Mada/Dr. Sardjito General Hospital, Yogyakarta, Indonesia; 5 Department of Global Health and Development, London School of Hygiene & Tropical Medicine, London, UK; 6 School of Tropical Medicine and Global Health, Nagasaki University, Nagasaki, Japan; 7 Faculty of Medicine, Universitas Udayana, Denpasar, Indonesia; 8 Department of Biostatistics, Epidemiology, and Population Health, Universitas Gadjah Mada, Yogyakarta, Indonesia; 9 ThinkWell.Global, Jakarta, Indonesia

**Keywords:** COVID-19, Cohort study, Health systems, Public Health, Tuberculosis

## Abstract

**Introduction:**

In Indonesia, a country with around 280 million people and the second-highest tuberculosis (TB) incidence rate in the world, the impact of the COVID-19 pandemic on TB care needs careful assessment so that future response strategies can be strengthened. We conducted a study comparing TB testing and treatment rates before and during the first 2 years of the COVID-19 pandemic in Indonesia, and the reasons for any disruptions to care.

**Methods:**

We conducted retrospective secondary data analysis and qualitative interviews in Yogyakarta and Bandung, Indonesia. Routine data on TB testing and treatment were sourced from the national TB information system operated by the Indonesian Ministry of Health. TB testing and treatment outcomes were compared between two time periods: pre-COVID (2018–19); and during COVID-19 (2020–21). In-depth interviews were conducted with patients and health workers to explore their experiences in accessing and providing TB services during the pandemic.

**Results:**

There was a 45% (21 937/39 962) reduction in the number of patients tested for TB during the pandemic compared with pre-COVID-19, while the proportion of TB tests returning a positive result increased from 12% (4733/39 962) to 50% (10 945/21 937). The proportion of TB patients completing treatment increased by 2.6% during the pandemic, yet the proportion cured and the number of patients successfully treated both decreased (by 7% and 4.4%, respectively). Our qualitative interviews highlighted several factors influencing TB service access and delivery, including fear of being diagnosed with COVID-19 during TB-related clinic visits, fear of COVID-19 exposure among patients and health workers, healthcare facilities prioritising COVID-19 over other services, and mandatory mobility restrictions affecting both patients and health workers.

**Conclusion:**

The COVID-19 pandemic impacted TB testing and treatment outcomes in Bandung and Yogyakarta. Policymakers should consider these findings in designing strategies to ensure TB services are maintained and supported during future health crises.

**WHAT IS ALREADY KNOWN ON THIS TOPIC**

- Several studies have investigated the impact of COVID-19 on tuberculosis (TB) care in Indonesia. Overall, these studies show the adverse impact of the pandemic on TB service delivery with substantial variation across districts. To our knowledge, this will be first mixed methods study, combining the strengths of secondary data analysis with in-depth interviews, to gain a deeper understanding of the impact of the pandemic on TB care from both a patient and health worker perspective.

**WHAT THIS STUDY ADDS**

- We found that during the COVID-19 pandemic in two Indonesian cities (Yogyakarta and Bandung), there was a decline in the number and proportion of people who were tested for TB, who completed treatment and who were successfully treated. A number of factors impacted patient access to TB services during the pandemic including mandatory mobility restrictions, fear of being diagnosed with COVID-19 and fear of contracting COVID-19 at healthcare facilities (HCFs). Barriers reported by health workers included a shift in clinic activities towards COVID-19, inability to conduct TB contact tracing and screening due to mobility restrictions, limits on the number of patients permitted to attend HCFs, reduction in TB treatment services to medication provision, and unwillingness to check patient sputum for TB diagnosis due to fear of contracting COVID-19.

**HOW THIS STUDY MIGHT AFFECT RESEARCH, PRACTICE OR POLICY**

- Our findings highlight the need for effective mitigation strategies to minimize pandemic-related disruptions to TB services in Indonesia including widespread public health awareness campaigns, integrated screening and case-finding for TB and COVID-19 (and other respiratory illnesses), and optimizing the use of telemedicine.

## Introduction

Indonesia has made significant progress in tuberculosis (TB) control, with a declining trend in incidence from 370 per 100 000 people in 2000 to 312 per 100 000 persons in 2019.^1^ However, TB remains one of the country’s top causes of morbidity,^2^ with 562 049 new cases notified in 2019.^3^ The COVID-19 pandemic put TB services under increased pressure, threatening to reverse years of progress in TB control.^4^

Indonesia has been hit hard by the pandemic, with 4.2 million confirmed COVID-19 cases and 140 000 deaths due to COVID-19 in 2021.^5^ The potential disruptions to TB services due to the pandemic in Indonesia are multifaceted and include reduced TB case detection, monitoring, evaluation and surveillance activities.^6^ Moreover, the pandemic led to the closure of healthcare facilities (HCFs), diversion of medical personnel toward COVID-19 prevention and treatment, reduced access to TB laboratory services and delays in follow-up visits for individuals with TB.^7–10^ Additionally, several studies of public HCFs in Indonesia have shown that investigation of presumptive TB cases decreased during the pandemic, along with TB case notification rates and TB treatment coverage.^11 12^ In contrast, a study of private HCFs reported no major changes in the quality of tuberculosis care due to the COVID-19 pandemic.^13^ This indicates that there are gaps in the direct experience of patients and health workers when accessing TB services during the pandemic.

Previous studies have revealed a range of ways in which the COVID-19 pandemic has disrupted TB programmes in Indonesia, mostly by using national cohort data.^11–14^ Far fewer studies have explored the perspectives and experiences of patients and health workers during the pandemic using mixed methods. To better understand the influence of the COVID-19 pandemic on the TB care cascade and the lived experience of patients and health workers, we compared TB testing and TB treatment outcomes before and during the pandemic and interviewed TB patients and health workers receiving/delivering TB care during the pandemic in two major Indonesian cities, Yogyakarta and Bandung.

## Methods

### Study design

Our mixed methods approach was based on a convergent parallel design where quantitative and qualitative data collection and analysis were conducted concurrently. Quantitative and qualitative findings were analysed separately and merged during the final interpretation phase. A stakeholder meeting was conducted at the end of the study, involving government representatives from the district, provincial and national levels to share and discuss both sets of data. This strategy was employed to enhance the trustworthiness of the data.^15^

### Study setting

This study is part of the DOMINO project, which aims to investigate the impact of the COVID-19 pandemic on TB and HIV services in Indonesia. It is supported by the United Kingdom National Institute for Health Research and UK Research Innovation through their Global Effort on COVID-19 Health Research funding scheme.^16^ We used secondary healthcare facility data from two cities: Bandung in the province of West Java (population 2.5 million) and Yogyakarta City in the Special Region of Yogyakarta (population 416 000). These cities were chosen due to their high burden of both COVID-19 and TB.

Indonesia experienced a sharp increase in COVID-19 cases during the second pandemic wave (Delta variant) which occurred in June–August 2021.^17^ Among the various government policies to control the spread of COVID-19 (see online supplemental table S1), the Community Activities Restrictions Enforcement (CARE) policy was implemented in areas with a large number of COVID-19 cases, including Yogyakarta and Bandung.^18–20^ CARE was designed to reduce COVID-19 transmission by restricting access and mobility to several key services including schools, restaurants and cafes, shopping malls, public transportation, recreational facilities, and places of worship.

10.1136/bmjgh-2023-014943.supp1 Supplementary data

While Indonesia has a wide network of public and private HCFs, only public HCFs were included in this study due to restricted data access. Yogyakarta has 21 public HCFs, consisting of 18 community health centres and three public hospitals.^21^ Bandung has 91 public HCFs, consisting of 80 community health centres, 10 public hospitals and one Centre for Community Pulmonary Health.^22^ All of these HCFs are required to provide TB testing and treatment based on national guidelines produced by the Ministry of Health (MoH).

### Study procedures

#### Evaluation study

We compared TB services between 2018 and 2021. The first 2 years (2018–19) were defined as the pre-COVID-19 period, and the last 2 years (2020–21) were defined as the during COVID-19 period.^23^ We used secondary data from the national TB information system developed and managed by the MoH specifically for drug-sensitive TB cases.^24^ TB testing (TB 04 register) and treatment (TB 03 register) data were derived from two different data sets (2018–21). Meanwhile, in 2018–19, we collected TB testing data manually because the TB information system did not collect this data.

Data extraction was conducted from January 2021 to January 2023. For TB testing and treatment analyses, we included those public HCFs that had complete data throughout the observation period (2018–21). There were 48 public HCFs (42 community health centres, 5 hospitals and 1 Centre for Community Pulmonary Health) that met the eligibility criteria. All data were accessed with permission from the City Health Offices (CHOs) in Bandung and Yogyakarta. TB testing and treatment analyses were conducted separately due to the lack of a common patient identifier in both datasets.

#### Qualitative study

Between February 2022 and June 2022, we conducted in-depth interviews with TB patients and health workers involved in the TB programme. Interviewees included purposively selected patients involved in the continuation phase of TB treatment and health workers who were able to provide insights into TB service delivery processes. Health workers were responsible for telephoning participants to briefly introduce them to the study and seek their permission for a member of the research team to contact them. Our researchers then arrranged a time to meet each patient at their regular HCF. Prior to each interview, the purpose of the research and topics to be discussed were explained to the participants who were then asked to provide their informed consent. Interviews continued until saturation of content was reached.^25^ We used a semi-structured interview guide to explore views and experiences related to the access and delivery of TB services during the COVID-19 pandemic, including the daily routines of health workers, perceived quality of care and medical supplies, and concerns about exposure to COVID-19 at HCFs. Interview guides were refined following pilot interviews in a neighbouring study location.

Interviews were held in a private room at the HCF to ensure confidentiality, lasted between 30 and 60 minutes, and audio-recorded with participant permission. All interviews were conducted in Bahasa Indonesia. The interviewers were native speakers of Bahasa with training in qualitative research methods.

### Data analysis

#### Evaluation study

The number of individuals tested and treated for TB was compared before and during the COVID-19 pandemic. TB testing and treatment data were analysed separately for each period due to data linkage problems. Data collected for each TB patient from the national TB information system included age, sex, healthcare facility level, TB regimen, TB diagnosis method (bacteriological or clinical), anatomical site of TB and treatment outcome. The TB testing dataset only included test results based on sputum testing.

In the national information system dataset, TB diagnosis was bacteriologically confirmed or established after clinical assessment.^26^ Bacteriologically confirmed cases were defined as individuals with a positive biological specimen by smear microscopy, mycobacteriological culture or Xpert MTB/Rif results (exclude rifampicin resistant), which has been approved for use in Indonesia since 2016. At the beginning of this study, utilisation of Xpert MTB/Rif was still limited to hospitals. In 2020, it began to increase to referral community health centres.^27^ Clinically diagnosed cases were defined as individuals who did not meet the bacteriological confirmation criteria but were diagnosed with active TB by a clinician. TB treatment outcomes were classified as cured, treatment completed, treatment failed, died, lost-to-follow-up or not evaluated—based on categories approved by the WHO.^26^ Definitions of these outcomes are shown in the online supplemental textbox S1.

In line with previous studies, we categorised TB treatment outcomes into two groups: ‘successful’ (cured and treatment completed) and ‘unsuccessful’ (treatment failure, died, lost-to-follow-up and not evaluated/referred out).^28 29^ Data were analysed using Stata V.17 (StataCorp, College Station, TX, USA). Continuous data were summarised using the median and IQR, and categorical data were presented as numbers and percentages. The association between COVID-19 era and testing positive for TB was evaluated using logistic regression adjusted for age, sex and healthcare facility. The associations between COVID-19 era and starting TB treatment, and between COVID-19 era and being successfully treated for TB, were evaluated using logistic regression adjusted for age, sex, TB regimen, TB diagnosis method, anatomical site of TB, HIV status, and healthcare facility. Statistical significance was defined as p<0.05.

#### Qualitative study

Audio recordings of the interviews were transcribed verbatim and analysed with NVivo Release 1.7.1 software to explore experiences with accessing and delivering TB services during the COVID-19 pandemic. Two researchers (SNSN and SDW) coded the data independently. Any coding disagreements were resolved through discussion with AP. The codes were then classified into categories and themes. A consensus meeting involving SNSN, SDW, YM, LPLW, ML, DB and AP was held to discuss and refine the categories and themes.

### Research ethics

The DOMINO study was approved by the ethics committees of Universitas Gadjah Mada Yogyakarta (KE/FK/1410/EC/2021), London School of Hygiene and Tropical Medicine (UK No 22829) and the University of New South Wales (HC200989). All ethics committees approved the waiving of consent for inclusion in our cohort study on the grounds that we were using de-identified secondary data that presented negligible privacy concerns. Prior to conducting the in-depth interviews, the study was explained to interviewees who then signed a consent form.

## Results

### TB testing

The cohort dataset included 61 899 observations of patients who underwent TB sputum testing. Compared with the pre-COVID-19 period, there was a 45% reduction in patients who underwent TB testing during the COVID-19 period (39 962 vs 21 937). Patient demographic characteristics at the time of TB testing were comparable to those in the pre-COVID-19 and the during COVID-19 period, as shown in table 1.

**Table 1:** Patient demographic characteristics in TB testing dataset

Variables	Pre-COVID-19	During COVID-19
Total number	39 962	21 937
Age (years)		
Median (IQR)	41 (25; 56)	40 (25; 55)
Age group		
<16	1780 (4.5)	1312 (6.0)
16–19	2532 (6.3)	1452 (6.6)
20–29	8136 (20.4)	4701 (21.4)
30–39	6186 (15.5)	3379 (15.4)
40–59	12 494 (31.3)	7197 (32.8)
>59	7726 (19.3)	3895 (17.8)
Missing data	1108 (2.8)	1 (0.0)
Sex		
Male	20 597 (51.5)	11 622 (53.0)
Female	19 309 (48.3)	10 315 (47.0)
Missing data	56 (0.1)	0 (0.0)
Healthcare facilities level		
Community Health Centre	17 233 (43.1)	7311 (33.3)
Hospitals*	22 729 (56.9)	14 626 (66.7)
Missing data	0 (0.0)	0 (0.0)

*Including the Centre for Community Pulmonary Health.

**Figure 1:**
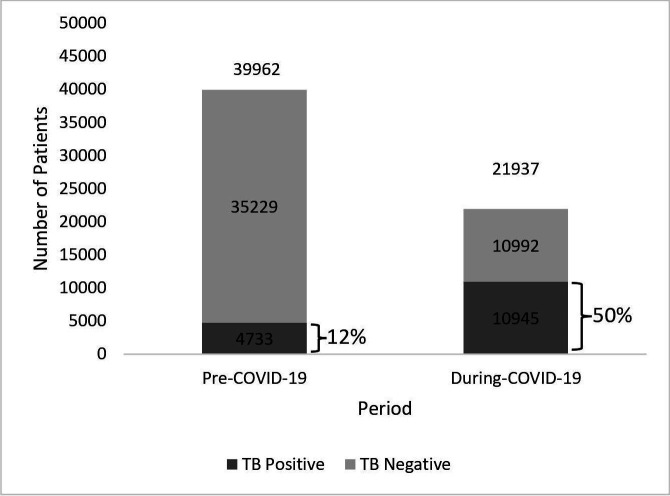
Number of TB testing in both cities.

During the pandemic, a higher percentage of positive test results were recorded than before the pandemic (50% compared with 12%) (figure 1). Adjusting for differences in population demographics and healthcare facility characteristics, the COVID-19 period was significantly associated with patients having a TB-positive test result (aOR=7.21, 95% CI [6.92 to 7.52]) (see online supplemental table S2).

### TB treatment

A total of 12 807 TB patients were either bacteriologically confirmed or clinically diagnosed. Table 2 shows that patient demographic characteristics at the time of TB treatment were similar pre-pandemic and during the pandemic.

**Table 2:** Patient characteristics in TB care

Variables	Pre-COVID-19	During-COVID-19
Total number	**7032**	**5775**
Age (years)		
Median (IQR)	28 (18–45)	31 (20–48)
Age group		
<16	1469 (20.9)	816 (14.1)
16–19	533 (7.6)	499 (8.6)
20–29	1669 (23.7)	1392 (24.1)
30–39	1075 (15.3)	881 (15.3)
40–59	1626 (23.1)	1566 (27.1)
>59	660 (9.4)	621 (10.8)
Missing data	0 (0.0)	0 (0.0)
Sex		
Male	3839 (54.6)	3148 (54.5)
Female	3193 (45.4)	2627 (45.5)
Missing data	0 (0.0)	0 (0.0)
TB regimen		
Category 1	4960 (70.5)	4421 (76.5)
Category 2	751 (10.7)	623 (10.8)
Other	1 (0.0)	11 (0.2)
Paediatric	1318 (18.7)	720 (12.5)
Missing data	2 (0.0)	0 (0.0)
TB diagnosis method		
Bacteriologically confirmed	3141 (44.7)	2685 (46.5)
Clinically diagnosed	3881 (55.2)	3090 (53.5)
Missing data	10 (0.1)	0 (0.0)
Anatomical site of TB		
Pulmonary	5518 (78.5)	4640 (80.3)
Extra-pulmonary	1514 (21.5)	1135 (19.7)
Missing data	0 (0.0)	0 (0.0)
HIV status		
Negative	1180 (16.8)	366 (6.3)
Positive	106 (1.5)	61 (1.1)
Not known	5746 (81.7)	5348 (92.6)
Missing data	0 (0.0)	0 (0.0)
Healthcare facilities level		
Community Health Centre	3489 (49.6)	2684 (46.5)
Hospitals*	3543 (50.4)	3091 (53.5)
Missing data	0 (0.0)	0 (0.0)

*Including the Centre for Community Pulmonary Health.

Among those starting TB treatment within our cohort, 614 (4.8%) were immediately referred to a HCF outside the cohort and 79 (0.6%) did not have any data on treatment outcome. TB treatment outcomes for the remaining 12 114 TB patients are presented in table 3. The proportion of patients retained in care during the COVID-19 pandemic was lower than in the pre-COVID-19 era (90.9% vs 93.6%, see online supplemental table S2). Table 3 shows that of those retained in care (n=11 188), the proportion of TB patients completing treatment increased by 2.6% during the pandemic compared to pre-pandemic (58.7% vs 61.3%) while the proportion achieving TB cure or completing treatment fell (84.6% vs 89.0%). In the adjusted analyses, we found that the COVID-19 period was associated with significantly lower odds of being retained in care (aOR 0.80 (95% CI 0.70 to 0.92)) and achieving TB cure or completing treatment (aOR 0.82 (95% CI 0.70 to 0.97)) (see online supplemental table S2). The proportion of TB patients who died on treatment was 2.3% pre-COVID-19 and 4.5% during the COVID-19 pandemic.

**Table 3:** TB treatment outcome

Variables	Pre-COVID-19	During-COVID-19
Total number*	**6509**	**5605**
Successful treatment		
Completed	3822 (58.7)	3437 (61.3)
Cured	1973 (30.3)	1304 (23.3)
Unsuccessful treatment		
Failed	150 (2.3)	104 (1.9)
Died	147 (2.3)	251 (4.5)
Lost-to-follow-up	417 (6.4)	509 (9.1)
Not evaluated/referred out	521 (7.4)	93 (1.6)

*Total of successful and not successful treatment

### Qualitative findings

**Figure 2:**
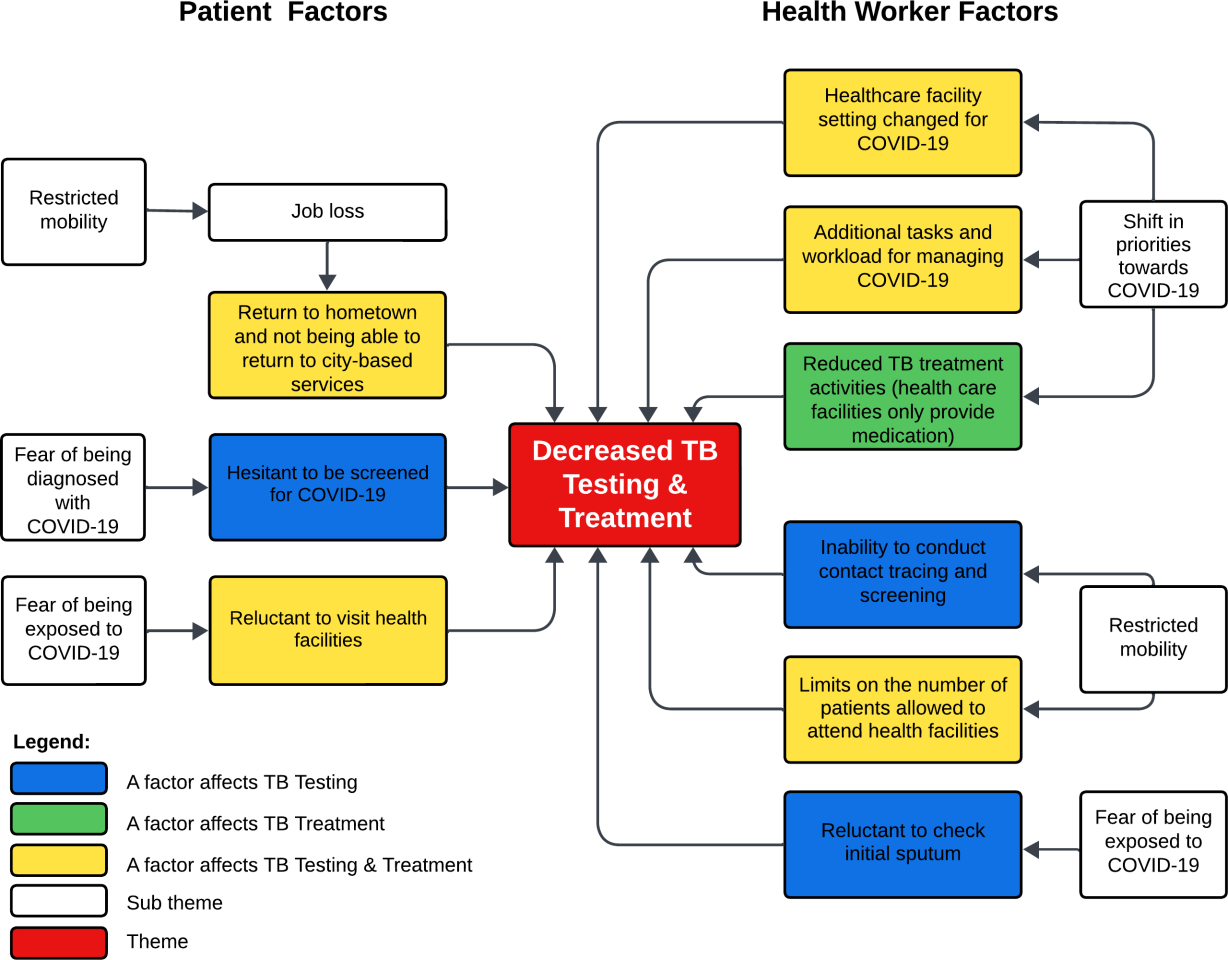
Barriers to the access and delivery of TB testing and treatment.

We interviewed 16 participants: eight TB patients and eight TB health workers from eight HCFs (online supplemental table S3). All TB patients (7 women, 1 man) were in the continuation phase (after the first 2 months) of treatment. All TB health workers were women with more than 10 years’ experience in the delivery of TB services (online supplemental table S4). Seven of the health workers were nurses, while one was a medical specialist (online supplemental table S4). Interviews revealed a range of barriers to the access and delivery of TB testing and treatment services during the COVID-19 pandemic (figure 2).

#### Patient factors

##### Restricted mobility

The CARE policy discouraged people from leaving their homes. This meant that many patients—especially those working in the informal sector such as domestic workers, home-based workers, street vendors and construction workers—lost their jobs and could not always afford transport and other out-of-pocket costs of seeking TB care. Moreover, many patients who lost their jobs during the pandemic and had to move back to their hometowns for financial reasons could not register for treatment with a new HCF, neither could they return for treatment at their original HCF because of the CARE policy.

From the patient’s perspective, there may be many who are economically affected. They have obstacles coming here because they do not have money. In this pandemic condition, they cannot eat; they do not have money to go to the community health centre. Maybe the impact is indeed on their finances but in the end, it does affect the treatment. – Informant 5, TB Health Worker

Especially in this pandemic, patients go back to their hometown and cannot come back here (healthcare facility) because of the Community Activities Restrictions Enforcement (CARE). So, there is indeed a decline in the number of patients receiving treatment. – Informant 5, TB Health Worker

##### Fear of being diagnosed with COVID-19

In the early phases of the pandemic, information and knowledge about COVID-19 were limited. Due to the similarity between TB and COVID-19 symptoms, TB patients were afraid of being diagnosed with COVID-19.

My husband does not want to go to the doctor and he does not want to go to the hospital (for TB care). In the past, when COVID-19 cases were high, he was afraid that he would be diagnosed with COVID-19 because he kept coughing. – Informant 3, Patient

One patient said that to avoid such a situation, they should take fever-reducing medication before coming to the healthcare facility.

Every time before going to the healthcare facility, I take Paracetamol first. Therefore, my body temperature drops when they check the temperature at healthcare facility. If the body temperature has dropped, then I will go to the healthcare facility because I am afraid of being misdiagnosed with COVID-19 if I have a fever. – Informant 2, Patient

Other patients said they would do a COVID-19 test before being tested for TB. However, long waiting times for COVID-19 test results, especially at the outset of the pandemic, meant patients often did not return for TB testing.

Now (during COVID-19 pandemic), when I go for treatment (during the pandemic), people who are coughing and who are sick are usually separated. There you will be swabbed first if you cough. If the swab is negative, continue with PCR. So, initially it was 1 door, now it is separated. – Informant 5, Patient

##### Fear of being exposed to COVID-19

Many patients were hesitant to visit HCFs because they feared being exposed to the COVID-19 virus and infecting themselves and their families.

I'm worried about being exposed to COVID-19 at the community health centre, even though I'm wearing a mask, but I'm afraid the mask will leak. – Informant 6, Patient

I do have concerns (of being exposed to COVID-19) because I have a child. What worries me is when I have to bring my child (to the healthcare facility). – Informant 1, Patient

Health workers confirmed that many TB patients were afraid to go to HCFs because they were afraid of contracting COVID-19.

Patients are indeed afraid of the hospital and at that time our patients also reduced. – Informant 2, Health Worker

#### Health worker factors

##### Shift in health service priorities towards COVID-19

During the COVID-19 pandemic, HCFs were rearranged to provide more physical space to manage COVID-19 patients. The additional tasks and workload of health workers also increased due to their involvement in COVID-19 contact tracing activities, the need to attend to COVID-19 patients, the replacement of colleagues who were self-isolating and additional tasks for COVID-19 prevention and care, including triage, swab tests and vaccine delivery. Furthermore, human resources allocated to CHOs and HCFs for the delivery of TB services were reportedly reduced during the pandemic.

At the beginning (of the COVID-19 pandemic) the pulmonary polyclinic at the hospital was also used for COVID-19 services. This affects TB because when COVID-19 escalated, we actually had a specific room for sensitive TB and a specific room for MDR TB, this then shifted. – Informant 3, Health Worker

The problem is that our time at home is taken up for tracing (COVID-19 patients). Sometimes, after we have traced, we have to make a report as well, we have to type it on the same day so it can be reported, because the data was requested. That is consuming our energy and time as well. – Informant 2, Health Worker

That is why when someone is under self-isolation, if it is a fellow nurse or colleague; it is automatic that someone (other health staffs) has to take care of (managing) the service. – Informant 8, Health Worker

Especially since the COVID-19 pandemic, you can say that the workload has increased. We have to be in the ILI (Influenza-Like Illness) clinic in the front to separate the infected from the non-infected. Those who swab, not to mention those who administer vaccines. So sometimes, I am alone, usually there are colleague who accompany me in TB polyclinic – Informant 5, Health Worker

In addition, at the height of the pandemic, there was a decline in the scope of TB services provided to patients. One health worker mentioned that TB patients were only provided with medication, while counselling and examinations were reduced or discontinued:

There are differences in TB services when COVID-19 cases rise, because many of us are infected with COVID-19, hospitals are being sterilized, and there have been closures of polyclinics. But for TB polyclinic, it cannot be interrupted, so the treatment is done outside (the building). Therefore, it is just providing medication. So, it is true that quality (of treatment) may decrease, but it just temporary, only when COVID-19 cases increase. – Informant 7, Health Worker

Sometimes, there are colleagues in counseling with patients; they feel afraid of being in contact for too long. So maybe that is why the counselling was accelerated. – Informant 7, Health Worker

##### Restricted mobility

As mobility restrictions were rolled out, most TB health workers reported that contact tracing and screening services were difficult to deliver, especially at the peak of the COVID-19 pandemic. When COVID-19 cases began to decline, TB health workers attempted to resume these services by training some voluntary health workers from the community to work with HCFs and monitor their activities using WhatsApp.

Especially when there was CARE or when the COVID-19 cases were so high, I could not move to the area to do TB contact tracing. When cases were high, our mobility was limited, so in the end we could not do tracing activities. - Informant 3, Health Worker

Sometimes together with the cadres (community health workers/CHWs), we conduct contact investigation. Honestly, we want to be active, we want to immediately investigate contacts, but it turns out that there is a COVID-19 red zone. Therefore, we are afraid; we do the monitoring by WhatsApp. Therefore, it was a bit hampered; the investigation was a bit delayed. – Informant 2, Health Worker

Health workers also mentioned that fewer patients with respiratory symptoms visited HCFs during the height of the pandemic. As a result, fewer patients were screened and tested for TB.

Patients who visit community health centre with complaints of coughing or TB symptoms are very rare. Patient visits during the pandemic have greatly decreased. – Informant 2, Health Worker

To curb the spread of COVID-19, the Indonesian government also restricted the number of patients permitted to attend HCFs. One health worker mentioned that before COVID-19, they accepted all patients coming to the community health centre, but during the pandemic, they only accepted patients from their catchment area.

The service hours change when there is a COVID-19 swab activity. To reduce the crowd in the registration room, the number of registrations was reduced. – Informant 1, Health Worker

The treatment success and lost-to-follow-up indicators were not achieved because when the patient arrived, the quota for treatment at the hospital had run out, so they went home again. – Informant 7, Health Worker

We only accept (patients) within the region. That is why many people are angry. Usually, we can receive patients from anywhere, now doctors limit patients to the area, and only patients who have JKN (national health insurance). – Informant 6, Health Worker

Similar to reports by TB health workers, patients also said they had to arrive early to register due to long queues and limited quotas for TB treatment.

So, before the pandemic, at the patient registration desk, when we arrived at 8 or 9 there was still a queue number. But during the pandemic, we have come early in the morning. At 6 o'clock we were already queuing in front of the door over there, there were already many people fighting over queue numbers because it was also limited. – Informant 3, Patient

##### Fear of being exposed to COVID-19

TB health workers and laboratory staff stated that at the beginning of the COVID-19 pandemic, they were very concerned about contracting COVID-19.

Yes, there are concerns (of contracting COVID-19), especially when the COVID-19 cases were peaking in June/July (2021). I was also affected in June-July by 2 weeks of self- isolation. Apparently, other health workers are also the same because every day they are exposed to those with COVID-19. Even though they were supported with food and vitamins. – Informant 8, Health worker

During COVID-19, my lab staff were concerned about airborne TB transmission. They did not want to check the initial sputum. I pity the patient who is suspected of TB. The laboratory staff wanted to check the sputum after the patient had taken the medicine for 2 months. – Informant 6, Health Worker

## Discussion

Our study found that the COVID-19 pandemic resulted in a 45% decline in the number of TB tests conducted. Despite this, the number of TB diagnoses increased. Among people diagnosed with TB, adjusted analyses found that the odds of successful treatment were lower in the COVID-19 period. The proportion of TB patients who died or were lost-to-follow-up also increased during the pandemic. Contributing factors reported by interviewees in this study included the prioritisation of staff and other resources towards COVID-19, mandatory mobility restrictions and a fear among patients and health workers of being infected with COVID-19.

Disruptions to TB testing during the COVID-19 pandemic have been documented in various studies conducted in Asia, including in India, Nepal and Indonesia.^11 12 30^ Consistent with our study, researchers in India found that despite a drop in TB testing, the proportion of TB-positive cases increased during the pandemic.^25^ The drop in testing is likely to reflect reduced access and availability of TB services including outreach services such as case investigation and contact tracing. The increase in the TB positivity rate during the pandemic could be associated with the accelerated implementation of infection control and the practical approach to lung health (PAL) strategy in HCFs. There are two main approaches used in PAL related to TB control: (1) standardisation of diagnosis and treatment and (2) coordination between health workers.^31^ Additionally, in 2020, Indonesia implemented GeneXpert as the main diagnostic tool for detecting TB cases, leading to enhanced detection and accuracy of TB-positive cases.^11 32 33^

We found a 44% decline in the number of children <16 years old receiving TB treatment during the COVID-19 pandemic (from 1469 to 816). Several studies have also reported a significant decrease in paediatric TB notifications during the pandemic.^34–36^ This might be a consequence of the government’s public appeal to postpone bringing children to HCFs unless it is an emergency,^37^ leading to delays in diagnosis and an underreporting of cases.^34^ Our qualitative data also shows that some parents decided not to bring their young children to HCFs because they feared they would contract COVID-19.Despite the necessity for comprehensive infectious disease surveillance, Indonesia lacks accurate and complete data on TB and COVID-19 cases. While the pandemic has revealed important weaknesses in routine TB surveillance activities, these problems are not new. The underestimation of TB testing, diagnosis and treatment has been an ongoing issue resulting from a variety of factors ranging from a shortfall in funding for TB surveillance through to implementation issues such as a lack of time and training among staff.^38^

Further, our study showed that a higher proportion of TB patients were lost-to-follow-up or died during the pandemic. Several factors hindered access to TB treatment during the pandemic including limitations in the number of patients who could visit HCFs, fear of contracting COVID-19 among patients and health workers, mobility restrictions for both patients and health workers, and economic constraints on TB patients. Patients and health workers often expressed a fear of contracting COVID-19 or having their TB symptoms diagnosed as COVID-19, which may be partly attributable to the perceived stigma associated with a COVID-19 diagnosis during the early phases of the pandemic.^36^ This is supported by many other studies documenting the increased stigmatisation of TB patients during the pandemic due for example to an overlap in symptoms.^39 40^ A reduction in TB treatment success during the COVID-19 pandemic is consistent with the findings from studies outside of Indonesia.^6 12 41–43^ It is possible the situation in Bandung and Yogyakarta could have been worse without implementation of the mitigation strategies including the provision of medication for longer durations,^44^ and the use of telemedicine or telehealth to provide information regarding TB treatment.^6^

Strengthening TB services is important to mitigate the staggering impact of pandemics such as COVID-19. One approach is more effective integration of TB screening and screening for other respiratory infections, ensuring basic health services are maintained despite constraints. This integrated model has the potential to help capture people with TB who may have been missed during the pandemic.^45 46^ In a similar vein, the implementation and expansion of integrated COVID-19 and TB case-finding services have also been proposed in the context of Indonesia.^47^

One of the main strengths of our study is its mixed methods approach, combining a large multicentre dataset with interviews involving health workers and patients from eight HCFs to help intepret and corroborate the quantitative findings. However, some limitations should be considered. The study was conducted in two large cities on Java Island. Generalising the study findings to other settings in Indonesia must consider potential differences in healthcare systems, cultural practices and infrastructure. Furthermore, because we only included patients from public facilities, our findings may not be representative of private HCFs, attended by approximately 48% of all TB patients in Bandung and Yogyakarta. We have used secondary data derived from the national TB information system, which was known as the Integrated Tuberculosis Information System (SITT) in 2018–19 and then re-named as the Tuberculosis Information System (SITB) in 2020–21. SITT did not collect testing data; thus, we collected those data manually from HCFs. This led to a small volume of missing data; however, it is unlikely this impacted our main conclusions. In addition, the introduction of active case finding in Yogyakarta in 2021 and Bandung in 2020 could have also influenced our TB testing results. Finally, for qualitative data, we did not distinguish between factors influencing TB testing and TB treatment, this may limit the usefulness of these findings for intervention and policy design. In addition, for the qualitative component of the study, we were unable to capture the views of those who were lost-to-follow-up pre-treatment or during treatment since we did not have sufficient resources to track these patients.

## Conclusion

Our study findings show that the COVID-19 pandemic significantly impacted TB testing and treatment in Bandung and Yogyakarta. Barriers to TB care included patients being fearful that their TB symptoms would be confused for COVID-19, reduced COVID-19 screening, concern among patients and health workers of being exposed to COVID-19, mobility restrictions and HCFs prioritising space, staff and other resources to COVID-19 over TB. Strategies such as integrated TB screening, increased active case finding and optimising the use of telemedicine could minimise future disruptions to TB services.

## Data Availability

The de-identified data are available upon reasonable request to the corresponding author and adhere to the Indonesian regulation on data sharing.
