# Supplementary material for: “I pity the TB patient”: a mixed methods study assessing the impact of the COVID-19 pandemic on TB services in two major Indonesian cities and distilling lessons for the future
Source: BMJ Glob Health. 2024 May 16;9(5):e014943. doi: 10.1136/bmjgh-2023-014943 (PMC11103193; doi:10.1136/bmjgh-2023-014943)
Supplement: Supplementary data [file bmjgh-2023-014943supp001.pdf]

Supplementary Materials

Supplementary Table 1 (Table S1). Policies regarding COVID-19 in Indonesia

Policy	Content	Institutions
Presidential Decree 9/ 2020	Task Force Unit for the Acceleration of the Corona Virus Disease 2019 (COVID-19)	Ministry of State Secretariat
Government Regulation 21/ 2020	Large Scale Social Restrictions (PSBB)	Ministry of State Secretariat
Campaign of 5M health protocol	Individual health protocol to implement wearing masks, washing hands, maintaining distance, avoiding crowds, and limiting mobility and interaction	Ministry of Health
Minister of Home Affairs 15/21	Community Activities Restrictions Enforcement (CARE/ PPKM)	Ministry of Home Affairs
Minister of Health Regulation 10/2021	Implementation of COVID-19 Vaccination	Ministry of Health

Supplementary Textbox 1 (Textbox S1). TB Treatment Outcome

Cured: A patient with pulmonary Drug Sensitive Tuberculosis (DS-TB) who was smear- or culture-negative in the last month of treatment and on at least one previous occasion.
Treatment completed: A DS-TB patient who completed treatment without evidence of failure but had no record of sputum smear or culture results in the last month of treatment, either because tests were not performed or because results were unavailable.
Treatment failed: A DS-TB patient who had a positive sputum smear or culture at month 5 of treatment or later.
Died: A TB patient who died for any reason between diagnosis and treatment completion.
Lost-to-follow-up: A DS-TB patient who did not begin treatment or whose treatment was interrupted for two months or more.
Not evaluated/Referred out: A DS-TB patient for whom no treatment outcome had been assigned. This includes cases that had been "transferred out" to another treatment facility, as well as cases where the treatment outcome was unknown to the reporting facility.

Supplementary Table 2 (Table S2). Unadjusted and adjusted associations between COVID-19 era and TB positivity, completing TB treatment, and TB treatment success

	TB positive (among 61,899 tested)					Retained in TB care (among 12,114 starting treatment)					Cured or completed TB treatment (among 11,188 retained in care)				
	**n(%N)**	**Univariate OR**	**p**	**Multivariate OR***	**p**	**n(%N)**	**Univariate OR**	**p**	**Multivariate OR^**	**p**	**n(%N)**	**Univariate OR**	**p**	**Multivariate OR^**	**p**
**COVID era**															
Pre-COVID-19	4,733 (11.8)	1.00		1.00		6092 (93.6)	1.00		1.00		5795 (95.1)	1.00		1.00	
During COVID-19	10,945 (49.9)	7.41 (7.12, 7.72)	<0.001	**7.21 (6.92, 7.52)**	**<0.001**	5096 (90.9)	0.69 (0.60, 0.78)	<0.001	**0.80 (0.70, 0.92)**	**0.002**	4741 (93.0)	0.68 (0.58, 0.80)	<0.001	**0.82 (0.70, 0.97)**	**0.022**

All models use logistic regression
*Adjusted for age, sex, and health facility
^Adjusted for age, sex, TB regimen, TB diagnosis method, anatomical site of TB, HIV status, and health facility

Supplementary Table 3 (Table S3). Characteristics of TB Patients

Informant	Age	Sex	Treatment phase	Education Level	City	Location
1	26	Male	Continuation phase	High school	Yogyakarta	Community Health Centre
2	27	Female	Continuation phase	University	Yogyakarta	Community Health Centre
3	47	Female	Continuation phase	High school	Yogyakarta	Hospital
4	34	Female	Continuation phase	High school	Yogyakarta	Community Health Centre
5	43	Female	Continuation phase	High school	Bandung	Community Health Centre
6	21	Female	Continuation phase	University	Bandung	Community Health Centre
7	56	Female	Continuation phase	High school	Bandung	Centre for Community Pulmonary Health
8	30	Female	Continuation phase	High school	Bandung	Community Health Centre

Supplementary Table 4 (Table S4). Characteristics of TB Health Workers

Informant	Age	Sex	Education	City
1	39	Female	Medical Doctor	Yogyakarta
2	51	Female	Bachelor's degree in Nursing	Yogyakarta
3	54	Female	Diploma in Nursing	Yogyakarta
4	47	Female	Bachelor's degree in Nursing	Yogyakarta
5	43	Female	Diploma in Nursing	Bandung
6	53	Female	Diploma in Nursing	Bandung
7	43	Female	Diploma in Nursing	Bandung
8	48	Female	Bachelor's degree in Nursing	Bandung
